# The Effect of Microcosm Biofilm Decontamination on Surface Topography, Chemistry, and Biocompatibility Dynamics of Implant Titanium Surfaces

**DOI:** 10.3390/ijms231710033

**Published:** 2022-09-02

**Authors:** Vanessa Sousa, Nikos Mardas, Dave Spratt, Iman A. Hassan, Nick J. Walters, Víctor Beltrán, Nikolaos Donos

**Affiliations:** 1 Periodontology and Periodontal Medicine, Centre for Host-Microbiome Interactions, Faculty of Dentistry, Oral and Craniofacial Sciences, Kings College London, Guy’s and St Thomas’ NHS Foundation Trust, London SE1 9RT, UK; 2 Centre for Oral Clinical Research, Centre for Oral Immunobiology & Regenerative Medicine, Institute of Dentistry, Barts and The London School of Medicine and Dentistry, Queen Mary University London, London E1 2AD, UK; 3 Microbial Diseases, Eastman Dental Institute, University College London, London WC1E 6BT, UK; 4 Materials Chemistry Centre, Department of Chemistry, University College London, 20 Gordon Street, London WC1H 0AJ, UK; 5 Natural Resources Institute Finland, Latokartanonkaari 9, 00790 Helsinki, Finland; 6 Clinical Investigation and Dental Innovation Center, Dental School and Center for Translational Medicine, Universidad de La Frontera, Temuco 4780000, Chile

**Keywords:** peri-implantitis, decontamination, therapy, UV-C, photodynamic therapy, titanium Brush, biocompatibility, XPS, EDX

## Abstract

Since the inception of dental implants, a steadily increasing prevalence of peri-implantitis has been documented. Irrespective of the treatment protocol applied for the management of peri-implantitis, this biofilm-associated pathology, continues to be a clinical challenge yielding unpredictable and variable levels of resolution, and in some cases resulting in implant loss. This paper investigated the effect of microcosm biofilm in vitro decontamination on surface topography, wettability, chemistry, and biocompatibility, following decontamination protocols applied to previously infected implant titanium (Ti) surfaces, both micro-rough -Sandblasted, Large-grit, Acid-etched (SLA)-and smooth surfaces -Machined (M). Microcosm biofilms were grown on SLA and M Ti discs. These were treated with TiBrushes (TiB), combination of TiB and photodynamic therapy (PDT), combination of TiB and 0.2%CHX/1%NaClO, plus or minus Ultraviolet-C (UV-C) radiation. Surface topography was evaluated by Scanning Electron Microscopy (SEM) and Laser Surface Profilometry. Surface function was analysed through wettability analysis. Surface chemistry evaluation of the discs was performed under SEM/Energy-dispersive X-ray spectroscopy (EDX) and X-ray photoelectron spectroscopy (XPS). Biocompatibility was tested with the cytocompatibility assay using human osteoblast-like osteosarcoma cell line (MG-63) cells. Elemental analysis of the discs disclosed chemical surface alterations resulting from the different treatment modalities. Titanium, carbon, oxygen, sodium, aluminium, silver, were identified by EDX as the main components of all the discs. Based on the data drawn from this study, we have shown that following the decontamination of Ti surfaces the biomaterial surface chemistry and topography was altered. The type of treatment and Ti surface had a significant effect on cytocompatibility (*p* = 0.0001). Although, no treatment modality hindered the titanium surface biocompatibility, parameters such as the use of chemical agents and micro-rough surfaces had a higher cytotoxic effect in MG-63 cells. The use of smooth surfaces, and photofunctionalisation of the TiO_2_ layer had a beneficial effect on cytocompatibility following decontamination.

## 1. Introduction

The use of dental implants in everyday clinical practice is increasing, and considered to be one of the most successful treatments for the restoration of missing teeth [1,2]. However, over the years, the prevalence of biological complications has been increasing [3] and peri-implant diseases have become a clinical reality [4]. Peri-implantitis is a plaque-associated pathological condition occurring in tissues around dental implants, characterised by inflammation and progressive loss of supporting bone [5]. Notably, peri-implantitis stands as a prevailing clinical challenge not only due to the complex and dynamic biofilm ecosystem colonising the implant surface and its interplay with the host, but also due to the burdensome decontamination process of the contaminated implant fixture and consequent biomaterial alteration [6]. 

Different non-surgical and surgical solutions have been advocated for the treatment of peri-implantitis [7,8,9,10,11]. The successful clinical management of peri-implantitis is based on the evaluation of composite therapeutic end-points, that correspond to disease resolution. These include the clinical presence of shallow pockets, without bleeding-on-probing (BoP) and/or suppuration, and the maintenance of radiographic bone levels [12]. However, current forms of treatment for peri-implantitis are often inadequate and may result in implant loss [4,13,14]. As there is no consensus on a gold-standard protocol of care, nor any universally accepted recommendations, the treatment of peri-implantitis remains a clinical challenge [4,9]. 

Notably, in the search of enhanced hard tissue integration, a number of implant surface modifications [1] have been implemented throughout the past half-century [5,15]. These different surfaces exhibit a range of Ra values, ranging from 0.10 µm (machined surfaces), to 6 µm (rough surfaces) [16]. Although only a few studies provided data on the susceptibility to the development of peri-implantitis due to implant surface topography [17,18,19,20,21], implants with modified surfaces may show a higher susceptibility to peri-implant disease re-infection [22]. Moreover, remnants of debris (organic or inorganic), or alterations of the implant surface [23] following a range of decontamination therapies, may trigger a number of soft and hard tissue healing responses [24]. Therefore, it seems plausible that protocols for surface decontamination may have different effects based on the macro- and micro topography of the surface [25]. Whilst the elimination of the bacterial pathogens and their remnants is vital for clinical stable implant outcomes, implant surface biocompatibility, initial severity of disease and defect morphology may influence the outcome for certain therapies [26]. 

The implant surface properties such as topography, chemistry, and biocompatibility, have been extensively investigated in early osseointegration and prevention of early implant failure [1,27,28,29,30]. However, the effect of decontamination treatment on these surface properties are still poorly understood. This research provides an evaluation of the changes of sole or combination decontamination protocols (Ti brush, photodynamic therapy, 0.2%CHX/1%NaClO, and UV-C irradiation) on the Machined (M) and Sandblasted, Large-grit, Acid-etched (SLA) implant surfaces, and their resultant biomaterial surface topography, wettability, chemistry and biocompatibility following the application of these decontamination protocols.

## 2. Results

Different disinfection protocols (Table 1) were evaluated to understand factors altering the Ti surface characteristics following disinfection of M and SLA Ti surfaces.

### 2.1. Viable Biofilm Quantification

#### 2.1.1. Machined Ti Surfaces 

In M surfaces the undisturbed microcosm biofilm (MC) group showed a total viable count of *anaerobic* spp. and *aerobic* spp. of 8.74log_10_ CFU/mL (95%CI: 8.51-8.97log_10_ CFU/mL) and 8.40log_10_ CFU/mL (95%CI: 8.17-8.63log_10_ CFU/mL) respectively. In comparison to the MC group, the anaerobic (3.48log_10_ CFU/mL) and *aerobic* (3.43log_10_ CFU/mL) spp. decreased significantly (*p* = 0.001) in the MC+. After mechanical disruption alone (T1) of the steady-state 30-day peri-implantitis biofilm, anaerobic *spp.* (2.84log10 CFU/mL) and *aerobic* spp. (2.82log_10_ CFU/mL) were present. The groups involving combination therapy (T2, T3) and either mechanical or combination therapy plus UV-C irradiation (T1+, T2+, T3+) were not significantly different from the control sterile groups (*p =* 0.0001), yielding undetectable live bacterial counts.

#### 2.1.2. SLA Ti Surfaces

*Anaerobic* spp. (8.93log_10_ CFU/mL [95%CI: 8.55–9.29log_10_ CFU/mL]) over-represented the viable log_10_ CFU/mL in the MC group. In comparison to the untreated 30-day steady-state biofilms (MC), overall, the anaerobic and *aerobic* spp. decreased by ca. 2.57log_10_ CFU/mL in the MC+ group. Following mechanical therapy alone the *aerobic* spp., and *anaerobic* spp. showed a decrease of 5.44log_10_ CFU/mL and 5.82log_10_ CFU/mL respectively in comparison to the MC group. The groups involving combination therapy (T2, T3) and either mechanical or combination therapy plus UV-C irradiation (T1+, T2+, T3+) were not significantly different from the sterile control groups (*p =* 0.0001), yielding undetectable live bacterial counts.

All autoclaved groups (control and experimental groups) yielded undetectable live bacterial counts.

### 2.2. Surface Topography, Wettability, Chemistry and Biocompatibility

#### 2.2.1. SEM Imaging Ti Surfaces before and after Decontamination

A qualitative assessment was conducted before and after employing a number of decontamination protocols on SLA and M substrata. SEM images of the SC group confirmed the evident different configuration of M and SLA surfaces.

Groups MC, T1, T2 and T3 showed similar qualitative surface characteristics in comparison to their UV-C irradiated counterparts (MC+, T1+, T2+, T3+, respectively). In addition, SC was similar to SC+, SA, and SA+. 

The SEM analysis of the MC group revealed a biofilm structure fully covering the Ti surface. It was observed that once the SLA and M surfaces are covered by a microcosm biofilm, it is not possible to differentiate between surfaces.

**Figure 1:**
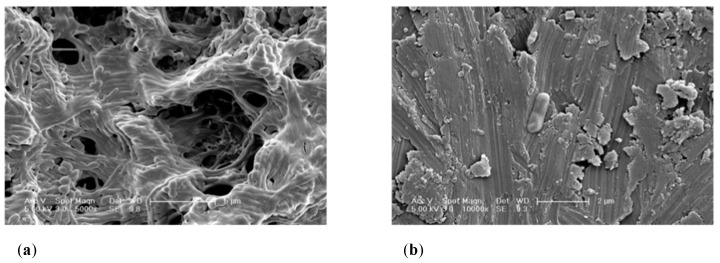
Scanning electron microscopy images showing (**a**) MC biofilm grown on top of Ti M substrata (scale bar = 5 µm, magnification 5000×), and (**b**) M Ti surfaces following disinfection employing T1 (scale bar = 2 µm, magnification 10,000×).

Following cleansing of the implant surfaces, remaining bacteria was found in all cases (Figure 1). It is evident that the use of a TiB for mechanical therapy will modify the surface topography of the Ti surface. For instance, SLA surfaces exhibited a combination of flattened areas combined with the typical SLA rough characteristics, whereas M surfaces exhibited scratches throughout the cleaned surface. Therefore, debris was present across the cleaned surfaces. Besides the organic bacterial remnants, this debris may correspond to detached abraded Ti particles or to the precipitation of crystals formed through the use of coadjutant chemical agents, such as PBS. 

#### 2.2.2. Surface Roughness

**Figure 2:**
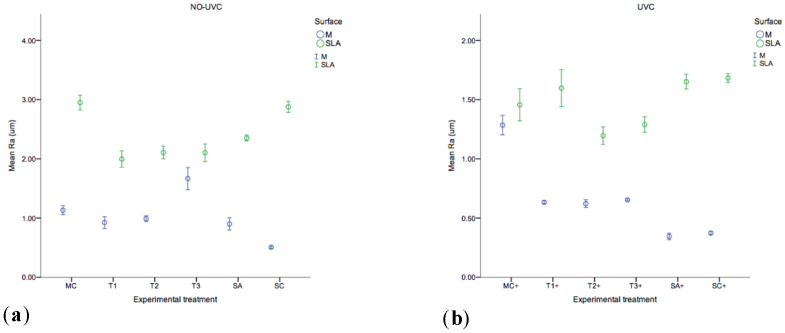
Surface roughness values of Machined and SLA topographies across (**a**) control (MC, SA, SC) and (**b**) experimental (T1, T2, T3) protocols −/+ UVC irradiation (*n* = 3). Each circle represents the mean and error bars the 95% CI.

The roughness (Ra) of each substrate before and after experimental therapy was analysed by laser profilometry with the results shown in Figure 2 (MC, T1, T2, T3, SA, SC, MC+, T1+, T2+, T3+, SA+, SC+). The SLA substrate (2.87±0.04um) was found to have the highest Ra value, which was, significantly rougher compared to M (0.50 ± 0.007um) (*p =* 0.000). Contrary to SLA surfaces, following mechanical therapy the Ra increased in M substrata (*p =* 0.000). Although Ra values decreased in mechanically treated SLA surfaces, these substrata remained with the highest Ra values (*p =* 0.000) before and after therapy. The UV-C irradiated groups T1+, T2+, T3+, SA+, SC+, showed significantly (*p =* 0.000) decreased Ra values, whilst non-UV-C irradiated groups (T1, T2, T3, MC, SA, SC) were found significantly rougher (*p =* 0.000).

#### 2.2.3. ddH_2_O Contact Angles

The photo-reactivity of the Ti discs was analysed by examining the ddH_2_O contact angle on the substrate following exposure to UV-C light before and after experimental decontamination of the substrata.

**Figure 3:**
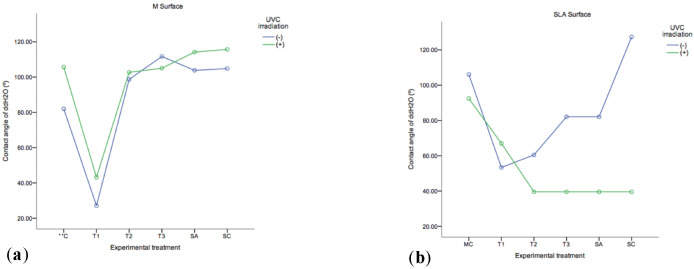
Contact angle of ddH_2_O on Machined (**a**) and SLA (**b**) substrate across control (MC, SA, SC) and experimental (T1, T2, T3) protocols +/− UVC irradiation. Each circle represents the mean and error bars the 95% CI (*n* = 3).

As shown in Figure 3 the most hydrophobic of the analysed samples was SC (SLA) (127.38 ± 0.323°). T1 (M) was shown to be the most hydrophilic group (27.05 ± 0.348°), followed by T3+ and T2+ (39.54 ± 2.19°), which were not significantly different from SC+ (i.e., sterile disc + UV-C irradiation) and SA+ (i.e., sterile disc + autoclaving + UV-C irradiation) (*p* = 1.00) in SLA substrata. UV-C irradiation had a higher effect on decreasing the contact angles in SLA in comparison to M substrata. In M substrata, the groups that included mechanical therapy and adjunct UV-C irradiation in their protocols (T1+, T2+, T3+) and groups T1 and T2, decreased their contact angles in comparison to control SA/+, SC/+ (*p* = 0.000).

#### 2.2.4. Surface Chemistry

**Figure 4:**
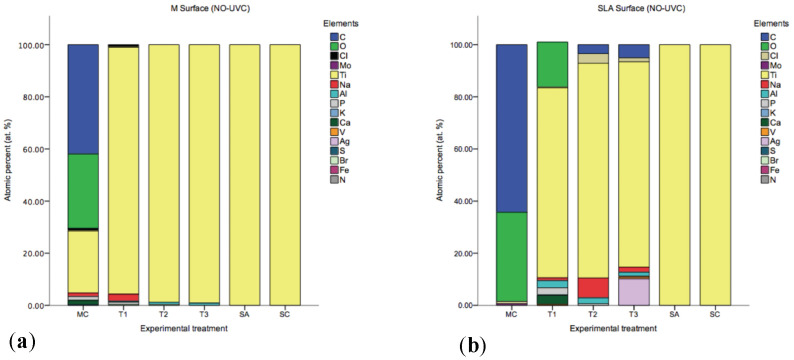
Results of SEM/EDX analysis, overview on mean atomic percentage (at. %) of elements on Machined (**a**) and SLA (**b**) implant surfaces before (MC, SA, SC) and after (T1, T2, T3) disinfection.

**Figure 5:**
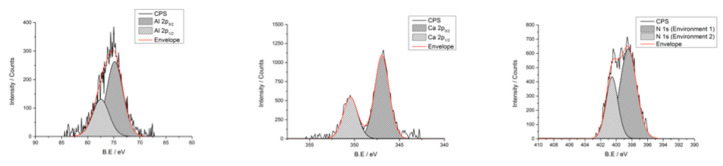
Effect of cleaning procedures on the Ti oxide layer (TiO_2_) surface properties. X-ray photoelectron spectroscopy high resolution scans detected peaks for Al(2p), Ca(2p) and N(1s). Samples were sputtered using a 2 kV argon ion gun; the sputtering rate was 1 angstrom per second.

Titanium (Ti), carbon (C), oxygen (O), sodium (Na), aluminium (Al), and silver (Ag) were identified by SEM/EDX as the main components of all the discs (Figure 4). Interestingly, small percentages of Nitrogen (N), vanadium (V), calcium (Ca), potassium (K) and chloride (Cl) were also detected, Figure 4 summarises the elements found. However, Ti was the only element found in the SC and SA groups. XPS analyses showed that cleaning procedures have an effect on the Ti oxide layer (TiO_2_) surface properties. X-ray photoelectron spectroscopy high resolution scans (Figure 5) detected peaks for Al(2p), Ca(2p) and N(1s).

#### 2.2.5. Cytocompatibility of Decontaminated Ti Surfaces

**Figure 6:**
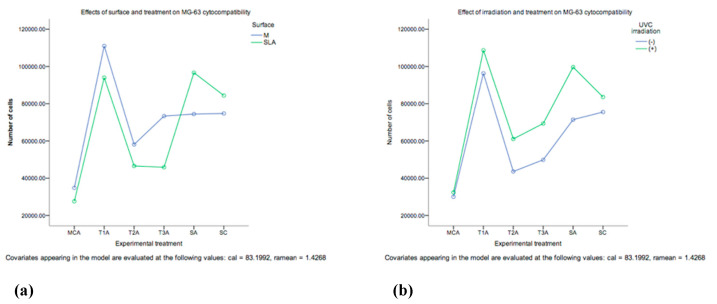
Evaluation of human osteoblast-like osteosarcoma cell line (MG-63) (**a**) proliferation across experimental treatments on Machined and SLA substrata (*n* = 3). The UVC-irradiated substrate (**b**) (in T1+, T2+, T3+, SA+, SC+) was shown to have a greater cell proliferation (*n* = 3) (*p* = 0.0001).

The cell proliferation of human osteoblast-like osteosarcoma cell line (MG-63) was examined over one week by employing the alamar blue assay. At day 1 SLA substrata exhibited higher cell proliferation. However, there was a higher cell proliferation in M substrata at a later stage on day 4 and 7. Overall, a higher number of cells was found at day 7 compared to day 1 (SLA and M surfaces). The proliferation of MG-63 seems to be affected by the employed experimental treatment (Figure 6). The lowest cell proliferation was found in the group MCA, whilst the combination therapies were not significantly different in terms of cell proliferation, the SA+ yielded the highest cell numbers (1.84 × 10^5^, 95% CI: 1.58 × 10^5^ – 2.09 × 10^5^) and was not significantly different from both SC+ and T1+. An interaction between treatment and surface was also observed (Figure 6). The proliferation of MG-63 seemed to be positively affected by UV-C irradiation. Groups T1A+, T2A+, T3A+, SA+, SC+, shown to have a greater cell proliferation in comparison to non-irradiated groups (T1A, T2A, T3A, SA, SC).

#### 2.2.6. SEM and CLSM Imaging of MG-63

**Figure 7:**
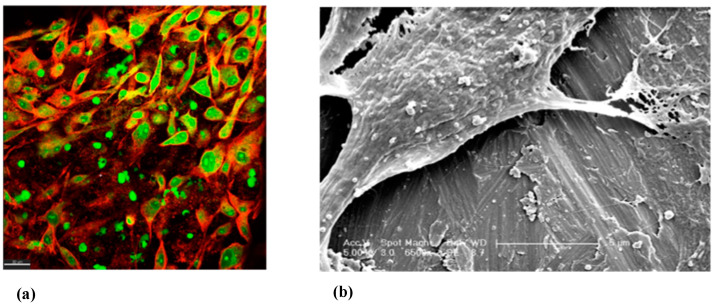
(**a**) Composite xy view CLSM image of MG-63 cells showing nucleus in green and actin filaments of the cytoskeleton in red grown on top of a disinfected M surface T2A+ group (scale bar = 30 µm). (**b**) SEM image of M surface T2A (scale bar = 5 µm, magnification 6500×).

The MG-63 cells were visualised under CLSM microscopy across all treatment modalities and at day 7 of incubation (Figure 7). In this experiment, the cells were found to grow on all treated surfaces. No qualitative morphological differences were observed.

## 3. Discussion

Although an important part of the peri-implantitis treatment pathway is the decontamination of the implant surface [7,9,23], the effect of decontamination protocols on dental implants derived from peri-implantitis sites is poorly understood. The evidence has reported that no surface decontamination method (e.g., Ti curettes, plastic curettes, Ti brushes, air-powder abrasives, laser application or implantoplasty) appear to yield superior outcomes to any other [26,31,32], and that there is no consensus on a standard protocol for the clinical treatment of peri-implantitis [33]. In the present research we have shown the associations of different in vitro decontamination protocols to the physico-chemical composition and the biocompatibility outcomes of M and SLA titanium surfaces before and after decontamination, and their consequent advantages and disadvantages are discussed below in light of their associations with specific outcomes. 

### 3.1. Viable Biofilm Quantification

A significant decrease (*p =* 0.001) in the CFU/mL viable counts were observed in all experimental groups in comparison to MC. Importantly, only groups MC+ and T1 had detectable live bacteria left behind. In the remaining groups T1+, T2-/+, and T3-/+ no live bacteria were recovered. Both surfaces yielded similar counts of viable bacterial remnants. The affinity of *Staphylococci* spp. to Ti surfaces has been documented in a number of studies [34,35]. Higher abundances of *S. aureus* have also been associated with peri-implantitis [36]. The success of antimicrobial therapy for peri-implantitis is often rated according to the bacterial load that can be found after treatment [37,38]. As previously mentioned, it is evident that mechanical disruption of the biofilm alone is not sufficient to eliminate the complex live bacterial consortia from the Ti discs and these findings are consistent with the current evidence [39,40]. Therefore, it is important that combination decontamination protocols are employed for the elimination of viable bacteria within peri-implantitis associated biofilms. 

### 3.2. Surface Topography, Wettability, Chemistry and Biocompatibility

#### 3.2.1. SEM Imaging and Surface Roughness of Ti Surfaces before and after Decontamination

This investigation showed by SEM imaging analysis of the experimental surfaces that bacteria were often observed positioned in the SLA crates and micropits, thus these irregularities seem to be forming an ideal niche for the live bacterial remnants. It is expected that these areas could act as reservoirs and germination nuclei for the bacteria and most importantly, they may be protected from the action of any employed chemical or antibiotic agents. This is fundamental to clinical practice, as our study showed that mechanical therapy of Machined surfaces also resulted in an increased number of Ti surface irregularities “scratches”, which according to our biofilm quantification outcomes, increased Ti surface irregularities will result in higher bacterial accumulation and facilitation of biofilm formation. 

The Ra value is one of the most common indicators to characterise surface roughness. However, it has been reported that the Ra parameter captures the surface topography in one direction and bias may be introduced by the profilometer [41]. In the present study, non-threaded Ti discs were used, thus enabling an accurate roughness evaluation. However, as Ti discs were employed instead of entire dental implants, the macro and microstructure of the screw models may be differently affected by the surface treatments as compared to the discs [42]. The use of mechanical therapy by means of a TiB resulted in the increase of Ra values for M surfaces. Conversely, and in agreement with a previous study [43] mechanical therapy of SLA surfaces resulted in a decrease of the surface roughness Ra parameters. The roughness and free surface energy of dental implant surfaces may influence the colonisation of bacteria [44]. However, it has been reported that a threshold value of Ra 0.2 µm does not impact the total amount of plaque of the colonizing bacteria [45]. In the present study, the lowest Ra value was observed in the SA+ (Ra = 0.34 µm) and SC+ (Ra = 0.37 µm) groups, followed by the group T2+ (Ra = 0.62 µm) in M surfaces. Therefore, the use of TiB will yield a surface roughness over the aforementioned threshold value, which may pose a benefit due to the reduction of surface roughness and the consequent elimination of organic debris. An alternative to smoother surfaces may be achieved by implantoplasty, however, that technique is out of the scope of the present study and warrants further investigation due to the side effects (e.g., implant fracture, release of Ti particles locally/systemically) that technique may convey [46,47].

#### 3.2.2. ddH_2_O Contact Angles

The present study investigated the photofunctionalisation of the TiO_2_ layer of the employed Ti discs by UV-C irradiation. This phenomenon, UV photofunctionalisation, converts the material from hydrophobic to super-hydrophilic (i.e., water contact angle of <5°) via surface irradiation. This process has been reported to be capable to improve the cellular response [48], as well as to reverse the effects of the time dependent degradation of biomaterial osteoconductivity [49]. It is assumed that upon UV irradiation, T4+ sites are converted to Ti3+ sites and in doing so, oxygen vacancies are formed at bridging sites which are more favourable for dissociative water adsorption [50,51]. In addition, UV photofunctionalisation has been reported to have a bactericidal and detoxifying effect [51,52,53], and to enhance cellular adhesion markers on various Ti substrata [54]. In the present study, the UV-C irradiated substrata, exhibited a decreased contact angle. Notably, SLA surfaces resulted in lower contact angles than M surfaces following irradiation. It seems that the use of TiB plus UV-C irradiation tends to decrease the contact angles and in consequence to a more hydrophilic implant surface. Furthermore, in non-UV-C irradiated samples, a decrease in contact angles was observed in TiB (T1) group in SLA and M substrata.

#### 3.2.3. Surface Chemistry

Additionally, after UV photofunctionalisation on the Ti, the charge of the substrate is changed from electronegative to electropositive, which enhances protein adsorption [54]. In the present study, the EDX analyses showed that UV-C irradiated surfaces had a higher Oxygen (O) at. % and a lower Carbon (C) at. % compared to the non-UV-C irradiated surfaces. A high C at. % was observed in the MC group, it is likely that the C source comes from the organic bacterial debris left behind after treatment. In addition, another important source of C may be introduced by the technique itself (for example, C content of the environment) and by the progressive deposition of hydrocarbons onto Ti surfaces [55]. Furthermore, a small Vanadium (V) at.% (i.e., 0.53) was observed, and this confirms the grade 5 according to Ti classification. In addition, remnants of Silver (Ag), Chlorine (Cl), and Sodium (Na) were observed in group T3 in SLA surfaces. Na was also present in groups T2 (TiB+PDT) and T1 (TiB) (SLA). Therefore, it is plausible that these inorganic remnants arise from the decontamination treatment modality employed and may provide a negative influence the re-osseointegration process [51]. 

#### 3.2.4. Cytocompatibility of Decontamination Ti Surfaces

Ti has been widely used as an implant material due to the biocompatibility and resistance to corrosion of its thin oxide layer [56]. After implant placement, the bone surrounding the implant interacts with the stable oxide layer found on the Ti surface and its alloys. This thin, durable, self-repairing oxide layer plays an important role in the osseointegration of the implant by mediating cell-surface interactions such as protein adsorption, cell adhesion and differentiation, and eventually bone formation and remodeling. The present study employed XPS analysis, in order to study modifications in the TiO_2_ properties. Notably, the element Ti was not detected in any of the samples. This may be due to the fact that samples were sputtered using a 2kV argon ion gun. The sputtering rate was 1 angstrom per second for 10 seconds, which allowed for a penetration of ca. 1 nm into the oxide layer. This finding may be explained by the assumption that Ti is located at a deeper layer and for instance, this may be further investigated by sputtering the samples for a longer period of time, and this finding warrants further investigation. However, as previously mentioned by means of EDX analyses, the presence of the element Ti was confirmed in all the samples, but not its state of oxidation, which may affect the biocompatibility of the titanium surface [51]. Further investigation is needed in order to assess the effect that this can cause in the re-osseointegration process.

It has been shown that re-osseointegration of previously contaminated implant surfaces is possible, and it is suggested that this mainly depends upon the surface of the implant, the types of decontamination techniques and bone regenerative materials used [57]. It seems that osteoblasts adhere more rapidly to rough surfaces [58]. However, some authors concluded that attachment is rather influenced by surface chemistry than surface roughness [59]. The results herein presented concur with this statement, and with previous studies that found that materials that have a similar roughness—and yet only differ in surface wettability- those that are super-hydrophilic have superior bioactivity [60,61].

It is important to point out that conflicting evidence has arisen in relation with autoclaving sterilisation of Ti surfaces. Some studies report no significant modifications of the oxide layer following autoclaving sterilization [62]. In contrast, other studies have found that the thickness, oxidation state, and composition vary with the sterilisation process used [50]. In the present study, an additional group SC (i.e., sterile disc not autoclaved) was included in the cell proliferation assay in order to compare, if any, significant differences with the group SA (i.e., sterile disc plus autoclaving). Additionally, in the surface chemistry analyses the SA group was included, so as to compare the surface chemistry with the SC group. No significant differences were found between these two groups by EDX and XPS analyses. 

The wettability or surface free energy of a substrate, is thought to play a pivotal role in the initial events that take place after implantation. In addition, no significant differences were found in terms of contact angles and Ra values, before and after sterilisation by autoclaving. However, the state of oxidation of the Ti oxide layer, as previously mentioned, was not determined and this aspect warrants further investigation. The latter is relevant given the effect of microbial biofilms have in inducing microenvironments that facilitate corrosion of Ti surfaces with subsequent release of Ti particles, which may synergise with tribocorrosion and facilitate implant failure. Host interactions to peri-implantitis biofilms and decontamination protocols warrants further investigation.

#### 3.2.5. SEM and CLSM Imaging of MG-63

In the present study, autoclaving sterilisation was performed previous to cell culture. The rationale behind sterilising the Ti surfaces previous to the proliferation assay is essentially due to the Alamar blue fluorescence/absorbance readings that may be influenced by live bacteria and yield inaccurate measurements. The SEM analyses, performed prior to the cytocompatibility assay, of treated Ti surfaces were found to harbour bacterial remnants. In this scenario, live bacterial cells will contribute to the cell number measurement in the AB assay. As such, live MG-63 and bacterial cells will both reduce resazuring (i.e., blue non-fluorescent compound) to resorufin (i.e., red fluorescence), thus both sources will contribute to such cell counts readings. In the present study, cell proliferation may have been enhanced by UV-C irradiation, time, type of treatment and surface roughness. Even though the MCA group was completely sterile, the fact that the lowest cell proliferation yield was found in this group, highlights the importance that the organic remnants left behind have in reducing the cell proliferation and warrant further investigation. UV-C photofunctionalised Ti surfaces had higher cells/mL yields. A higher amount of MG-63 cell was found at day 7 in comparison to day 1. Surface roughness seems to play a role only at day 1, when SLA surfaces had a higher cell/mL yield. Interestingly, at days 4 and 7 M treated surfaces had a higher cell proliferation than the SLA treated surfaces.

The group T1A+ had the highest amount of cells/mL and there were no significant differences between T2A+ and T3A+. It is hypothesised that these results were related mainly to the surface chemistry, since the inorganic remnants of the chemical substances used for contamination may have caused a delay in the cell proliferation. However, these elements did not seem to have altered the surface biocompatibility. Bone cells may be sensitive to the morphology of the material, leading to differences in their shape, orientation and adhesion [42,55]. However, in the present study, MG-63 cells were able to grow on all treated surfaces and no morphological differences were observed at day 7 of incubation. 

## 4. Materials and Methods

### 4.1. Microcosm Biofilm Growth

Biofilms were grown in a Constant Depth Film Fermentor (CDFF) peri-implantitis biofilm model as previously described [6]. The CDFF was inoculated with 500 mL of artificial saliva containing 1 mL aliquot of a pooled stock of human whole saliva. Microcosm biofilms were grown over commercially pure Machined (Sa = 0.40 μm) and SLA (Sa = 1.79 μm) Ti discs (grade IV; ASTM F 67; Institut Straumann AG, Basel, Switzerland) of 5 mm in diameter and 1 mm of thickness were used. Ethical approval of the protocol for human whole saliva sample collection and experimental research was provided by UCL Research Ethics Committee approval (1364/001).

### 4.2. Microcosm Biofilm Disruption

The different decontamination treatment modalities that were used in test groups and the positive (MC) and negative (SC, SA) controls are shown in Table 1. The specific decontamination protocols are summarised below.

#### 4.2.1. T1: Mechanical Disruption with Ti Brush

Mechanical disruption of the biofilms was performed by means of a titanium coated brush (TiB) (TiBrushTM Straumann; Basel, Switzerland) with a shank of 10mm in length and a titanium bristle at the end, fitted to a slow speed NSK (Nakanishi Inc., Tochigi, Japan) contra angle oscillating hand-piece (ER1 6i 16:1). The mechanical decontamination was carried out at a speed of 900 rpm, for 60 seconds, using a unidirectional movement across the disc. For each disc, a new TiB was used with a force of 0.3–0.5N. To avoid any contact on the surface of interest, the Ti disc was inserted into a custom-made disc holder (Staumann, Basel, Switzerland). This allowed surface decontamination without any interferences from any instrument. During the use of the TiB, the surface of the disc was simultaneously irrigated with 0.9% Phys. NaCl. Finally, the discs were rinsed 3 times, by dipping them into a well-plate containing 0.9% Phys. NaCl.

#### 4.2.2. T2: TiB + Photodynamic Therapy

Photodynamic therapy—PDT (PeriowaveTM, Vancouver, BC, Canada [λ 660–675 nm, 11 mW]), was used following the mechanical treatment of the Ti disc surfaces by the use of the TiB for 60s (as described above). One mL of photosensitiser (PS) consisted of 3,7-Bis (dymethyl-amino) phenazathionium chloride trihydrate (methylene blue) at the concentration of 0.005% (*w/v*) was added to the Ti disc, making sure that the entire surface was covered and left in situ for 60 s prior to irradiation [63]. This allowed sufficient uptake of the photosensitiser by the targeted microorganisms, and for the optimal action of the laser. A uniform light distribution was emitted from the tip of the pulsed diode soft laser (PeriowaveTM, Vancouver, BC, Canada) at a fixed distance of 10 cm from the disc. Static irradiation (25 mW/cm^2^) for the entire disc was completed in 60 s. The methylene blue was removed by dipping the disc into 0.9% Phys. NaCl (PBS), and repeatedly in a fresh new 0.9% Phys. NaCl to ensure removal of the photosensitiser until the disc was free of all visible methylene blue residues.

#### 4.2.3. T3: TiB + Chemical Agents

Chemical decontamination was performed following the mechanical treatment of the Ti disc surfaces by the use of the TiB for 60 s (as described above). Thereafter 1 mL of 1% NaOCl was applied for 60 s, after which the Ti disc was dipped in a sterile well-plate with 2 mL of a solution of 0.9% Phys. NaCl, and later in 1mL of 0.2% CHX for 60 s and before being rinsed.

#### 4.2.4. MC+, SC+, SA+, T1+, T2+, T3+: Ultraviolet Irradiation C

UV-C irradiated discs shown with a + symbol in Table 1 received a source of standardised UV-C light (4.2 mW/cm^2^, λ 254 nm, 230 V, 50 Hz, ca. 30 °C at Ti surfaces) at 10 cm from the source and centred. The irradiation installation was performed inside an opaque chamber, in order to prevent interference from the room or daylight, or cause any damage to users.

### 4.3. Effect of Therapies on Viable Cell Counts

The biofilm composition and bacterial remnants were investigated in the control and treatment groups before and after treatment across different decontamination strategies and substrata on SLA and M Ti surfaces with non-selective culture media analyses. Aerobes were cultured by plating the dilutions onto columbia blood agar (CBA [LabM, Lancashire, UK]). Anaerobes were isolated on to fastidious anaerobe agar (FAA [LabM, Lancashire, UK]).

### 4.4. Effect of Therapies on Surface Topography, Wettability, Chemistry and Biocompatibility

The detailed methodology is included in the supplemental file (Supplementary Materials). In brief, qualitative images were taken before and after treatment (Table 1) employing Scanning Electron Microscopy (SEM) (JEOL JSM 5410LV, Hertfordshire, UK) (Supplementary Materials). 

Surface topography (Proscan 1000, Scantron Ltd., Somerset, UK [laser KL131A, resolution {z} 0.02 μm]), and wettability (CAM 200, KSV Instruments, Biolin Scientific, MD, USA) were assessed (Supplementary Materials). 

The effect of the different decontamination protocols on surface chemistry (Thermofisher Scientific K-Alpha XPS system with Avantage software and JEOL JSM-6301F; Oxford Instruments INCA EDX) were tested (Supplementary Materials). 

Biocompatibility (MG-63, European Collection of Cell Cultures, Porton Down, UK), was evaluated (Supplementary Materials). Following the cytocompatibility assay, SEM and CLSM images (Bio-Rad Laboratories Ltd, Hemel Hempstead, Hertfordshire, UK) of MG-63 cells were obtained (Supplementary Materials).

### 4.5. Statistical Analyses

The number of colony forming units per biofilm was determined (i.e., CFU/biofilm = NCFU × DF × 50—where NCFU is the number of colonies, DF is the dilution factor and 50 is the inverse of volume plated). The means, SE (the SD of a statistic) of means and 95% CI of log_10_ CFU/biofilm bacterial counts [64] of the experimental groups (Table 1). Then, the analysis of residuals was performed and assumptions were satisfied only after the log_10_ transformation of the count data (CFU/biofilm). Thus, log10 CFU/biofilm was used to perform parametric statistics. Univariate general linear model (mean predicted values) and multiple comparisons with Bonferroni post-hoc corrections were performed to compare the log_10_ transformed bacterial counts per mL of each sample characteristics (i.e., control and experimental groups) and by surface characteristics. Then, differences in terms of log_10_ CFU/biofilm between surfaces were performed first with a two-way univariate analysis of variance in order to test surface and treatment as main effects and whether interactions were present. 

Linear regression analysis was performed as a first step in order to test whether a number of variables (day, therapy, UV-C and surface characteristics) were influencing the cytocompatibility response (i.e., number of cells/mL). Since, these variables resulted to be influencing the cytotoxicity response, then, a repeated measures ANOVA multilevel model 4-way interaction was performed in the cell proliferation experiment to compare the variables across time and at different levels (*p* < 0.0001).

Descriptive statistics were performed for the changes observed in the surface topography, and wettability. Surface chemistry was analysed through SEM/EDX INCA software to process the acquired data, then means ± SD of atomic percentage (at. %) were compared. XPS data scans were modeled using CASAXPS software and relevant descriptive graphs were included.

Qualitative analyses were undertaken for the changes observed in SEM and CLSM images across surfaces and treatments.

## 5. Conclusions

The data presented in this in vitro study suggest that titanium surface biocompatibility to MG-63 cells was not hindered by the use of TiB, combination of TiB and photodynamic therapy, or combination of TiB and 0.2%CHX/1%NaClO. 

It was demonstrated that:

1. Bacterial debris will prevail after mechanical and combination therapy.
2. Photofunctionalisation of the Ti surface with Ultraviolet-C (UV-C) radiation appears to have a positive effect in the Ti surface biocompatibility. MG-63 cell proliferation was enhanced by photofunctionalisation of the Ti surface by the use of UV-C irradiation, time, type of treatment and type of surface. The outcomes of this cytocompatibility study favours decontamination of smooth surfaces, and photofunctionalisation of the TiO_2_ layer.
3. All decontamination protocols induced modifications of the Ti oxide layer surface properties. Therefore, it is plausible to focus future research on developing decontamination protocols that facilitate the re-establishment of a biocompatible state of the implant surface chemistry.
4. Successful treatment for peri-implantitis should integrate a comprehensive approach, including not only the elimination of live bacteria, but also the bacterial debris, and taking into account the Ti surface biomaterial alterations, re-contamination of implant surfaces, and host interactions. Therefore, decontamination protocols for titanium (Ti) surfaces should be carefully chosen by the clinician.

## Figures and Tables

**Table 1:** Distribution of the experimental and control groups.

Control Groups *	Experimental Surface Disinfection Protocols * ^^ a^		
Biofilm (MC) ^a^	TiB (T1)	Irradiation ^c^	TiB+ (T1+)
Sterile (SC)	TiB+PDT (T2)		TiB+PDT+ (T2+)
Sterile autoclaved (SA)	TiB+CA (T3)		TiB+CA+ (T3+)
			Biofilm+ (MC+)
			SA+ (SA+)
			SC+ (SC+)

* Machined and SLA substrata were used in all groups (*n =* 3). ^^^ For the cytocompatibility assay the same control and experimental protocols were used following steamed heat autoclaved (A) at 121 °C/15 min. ^a^ Intact 30-day steady-state biofilms were used. ^c^ Exposure for 60 min to a source of standardised UV-C irradiation prior to testing.

## Data Availability

The datasets used and analysed in this study are available from the corresponding author upon request.

## Supplementary Materials

The following supporting information can be downloaded at: https://www.mdpi.com/article/10.3390/ijms231710033/s1.
