# Supplementary material for: The Effect of Microcosm Biofilm Decontamination on Surface Topography, Chemistry, and Biocompatibility Dynamics of Implant Titanium Surfaces
Source: Int J Mol Sci. 2022 Sep 2;23(17):10033. doi: 10.3390/ijms231710033 (PMC9456268; doi:10.3390/ijms231710033)
Supplement: Supplementary file 1 [file ijms-23-10033-s001.zip › ijms-1870724-supplementary.pdf]

Article

The effect of microcosm biofilm decontamination on surface topography, chemistry, and biocompatibility dynamics of implant titanium surfaces

Vanessa Sousa[1]*, Nikos Mardas[2], Dave Spratt[3], Iman A. Hassan[4], Nick J. Walters[5], Víctor Beltrán[6], Nikolaos Donos[2]

Supplementary Materials (SM):

Effect of Therapies on Surface Topography, Wettability, Chemistry and Biocompatibility:

Scanning Electron Microscopy (SEM) imaging of the experimental and control groups

SEM images were taken from either biofilms or decontaminated discs, to study the morphological characteristics of the bacteria, and the Ti surfaces characteristics (pristine and modifications induced by decontamination protocols) and bacterial remnants across a number of experimental conditions. Briefly, biofilms and decontaminated discs were carefully submerged in ultrapure high quality water (UHQ water) to remove any unattached or loosely associated bacteria. Then the suspension was removed from the wells and replaced with 4% gluteraldehyde with 0.1M cacodylate buffer (Agar Scientific, Stansted, Essex, UK) for 24 h at 4°C. Afterwards, the glutaraldehyde fixative was replaced with a graded series of ethanol (EtOH) solutions at the concentrations of 20%, 50%, 70% and 90% and 100% (3 times) for 10 min each so as to dehydrate the specimens. The final concentration of EtOH was replaced with hexamethyldisilazane (HMDS; TAAB Ltd, UK) for 2 min. The specimens were then left to dry overnight in a desiccator. The discs were mounted onto individual aluminium SEM specimen stubs (Agar Scientific, Stansted, Essex, UK) and sputter-coated with gold/palladium by means of a Polaron E5100 coating device (Polaron CVT, Quorum Technology, UK). The analysis of the discs was done in triplicates using a SEM JEOL JSM 5410LV (JEOL UK, Hertfordshire, UK) using various magnifications at an operating voltage of 10 kV.

Surface Topography and Wettability

The surface topography of each Ti substrate was analysed by laser profilometry (Proscan 1000, Scantron Ltd., Somerset, UK [laser KL131A, resolution {z} 0.02 um]) where a total area of 6.25 mm2 was examined on each surface (n= 3). Surface texture parameters were then calculated in the form of the arithmetical roughness average – Ra (i.e. selecting ten different areas on the scan plot per sample [top, middle, bottom –left and right, and random locations]) and the amplitude parameter average roughness –Sa (i.e. complete 3D surface evaluation per sample), values using Proscan software.

To measure surface wettability of the non-UVC and UVC treated Ti discs contact angle measurements were performed using an optical contact angle meter (CAM 200, KSV Instruments, Biolin Scientific, MD, USA) by employing 2 uL of ddH2O droplets on (n= 3). The left and right sides, and mean contact angles were calculated using the CAM 200, KSV software.

Surface Chemistry

Energy-dispersive X-ray spectroscopy (EDX) was carried out on a JEOL JSM-6301F Field Emission instrument with acceleration voltage ranging from 20 kV. Sample preparation involved cutting the films down to coupons 10 mm x 10 mm or less and sticking them on stainless steel holders using conductive carbon tape. Contacts from the top of the film to the carbon tape were made using a solution of silver paint. Once dried the samples were coated with a fine layer of carbon to avoid charging.

X-ray photoelectron spectroscopy (XPS) was carried out on Thermofisher Scientific K-Alpha XPS system with Avantage software. The base pressure in the chamber was 1x10-8 Torr. Samples were sputtered using a 2 kV argon ion gun, the sputtering rate was 1 angstrom per second (calibrated using a SiO2 standard and a relative sputter factor of GaAs of 1.3). High-resolution scans were

recorded for Al (2p), Cl (2p), Br (3d) Ca (2p), Mg (1s), P (2p), Ti (2p), V (2p), O (1s) and C (1s). The peaks were modelled using CASAXPS software with binding energies calibrated to carbon (284.5 eV).

Surface Biocompatibility

Human osteoblast-like osteosarcoma cell line (MG-63, European Collection of Cell Cultures, Porton Down, UK) was cultured under standard conditions (37 °C, 95% air, 5% CO2, 95 % relative humidity) in Dulbecco's modified Eagle medium (DMEM, Gibco, Life Technologies, Paisley, UK) supplemented with 10 % foetal bovine serum (Gibco) and 1% penicillin/streptomycin (PAA Laboratories, GE Healthcare, Chalfont St. Giles, UK).

Since it has been reported that the presence of live bacteria will interfere during cell culture and cytocompatibility assays, autoclaving of the Ti discs (for 121°C/15 min [Steam Heated RSC Priorclave, UK]) was performed prior to the cell proliferation assay (in experimental and control groups), a sterile control Ti substrata without autoclaving was also included during the experiment. The presence of live bacteria after autoclaving was investigated by culture media analysis (CBA and FAA agar). After sterilisation of the Ti discs, they were placed in ultra-low attachment 96 well plates (Corning, NY, USA). MG-63 were seeded at a density of 30,000 cells/cm2 on top of the specimens (n = 3) in 100 µL cell culture medium and incubated at 37 °C. A tissue culture plastic (TCP) control consisted of cells plated in a typical treated 96 well plate (Corning). Cytocompatibility was then assessed after 1, 4 and 7 days in culture, using 10% resazurin (alamarBlue Cell Proliferation Assay, AdB Serotech, Bio-Rad Laboratories Inc., Hemel Hempstead, UK) in cell culture medium, in accordance with ISO 10993-5:2012 [1]. After incubation at 37 °C for 1–2.5 h, once sufficient colour change had been observed, fluorescence (excitation: 560 nm; emission: 590 nm) was measured (FLx800, BioTek, Potton, UK). Standard curves were obtained by seeding a range of cell concentrations in 96 well plates, 2 h prior to each assay.

CLSM Imaging of MG-63 Cells

The actin filaments of the cytoskeleton of MG-63 cells (used to test cytocompatibility as it will be explained in section 2.4.6) were stained with F-Actin staining (Alexa Fluor 488 nm phalloidin, Invitrogen, UK) and counterstained (i.e. to stain the nucleus) with propidium iodine (BD, Biosciences, UK) (A.3.2) and an BX51 stereomicroscope (Olympus Ltd, Southall, UK) in conjunction with a Bio-Rad Radiance 2100 laser scanning system (Bio-Rad Laboratories Ltd, Hemel Hempstead, Hertfordshire, UK) was used to image the MG-63 cells using a LUMPlanFI x40 water immersion objective lens with a numerical aperture of 0.8 and a 2x digital zoom. Three images were obtained from each disc from random locations and visualised at different magnifications. This experiment was performed in order to visualize the MG-63 cells morphology and confirm the coverage of the substrata after cytocompatibility analyses. The acquired images were processed with ImageJ 1.46r.
